# The Nature of the Neurotic

**Published:** 1916-06

**Authors:** 


**The Nature of the Neurotic.** Philip Kilroy of Springfield, in a letter to the Boston M. & S. Jour., Oct. 14, compares Adler's description of the neurotic (Adler being in his estimation a filth-free disciple of Freud) to the description of clay in a proprietary compound as "levigated anhydrous argilaceous mineral matter." He states that "non-rainbow chasers" recognize that "the neurotic is just a plain damn fool, of varying degree, sometimes permanently, sometimes temporarily, unable to withstand or control the multiple stimuli of life, be they endogenous or exogenous."

